# Feasibility of integrating HIV testing into local youth development p rogrammes in Cameroon

**DOI:** 10.11604/pamj.2018.29.189.15313

**Published:** 2018-04-02

**Authors:** Cavin Epie Bekolo, Tatiana Danielle Yimdjo Fogue, Thomas D’Arcy Williams

**Affiliations:** 1 Department of Public Health, University of Dschang, PO Box 67 Dschang, Cameroon; 2 Centre Médical d’Arrondissement de Baré-Bakem, BP 628 Nkongsamba, Cameroun; 3 Peace Corps Cameroon, Yaoundé, Cameroun

**Keywords:** HIV testing, youths, Cameroon

## Abstract

Many young people do not know their HIV status in sub-Saharan Africa where it is estimated that only 10% of young men and 15% of young women know their HIV status. In a rural community in Cameroon, we present a collaborative project that has successfully nested HIV testing for youths into a youth life skills development programme run by youths themselves with support from various local government services and private organisations. We tested 2024 clients, including 1623 (80%) aged ≤35 years, of whom 839 (51.7%) boys and 784 (49.3%) girls. The number of young people becoming aware of their HIV status for the first time was 1256 (77.4%). We urge HIV programmes to be inspired by this example that made it easier for more young people to know their HIV status.

## Introduction

HIV is the leading cause of death among adolescents in Africa. Yet, HIV testing among youths is very low in sub-Saharan Africa where it is estimated that only 10% of young men and 15% of young women (15-24 years) know their HIV status. Discrimination, poverty, lack of youth services, geographical inaccessibility, inequality, repressive behaviour of parents often prevents young people in rural areas from accessing HIV testing, care and support services [1]. Only 9% of Cameroonian youths were reported to be knowledgeable about all HIV prevention strategies and approximately 28.5% were aware of their status [2,3]. In the rural community of Baré-Bakem, according to a preliminary community needs assessment conducted by a US Peace Corps Volunteer, over three out of four young persons were not aware of their HIV status. Approaching the commemoration of World AIDS Day 2017 in Cameroon, we organised a high-impact, youth friendly and collaborative project to promote youth testing in Baré-Bakem.

## Methods

From 28 November 2017 to 6 December 2017, a multi-sectoral project was organised in the locality of Baré-Bakem to promote youth welfare, with local actors from the Cameroonian ministries of public health, youth and civic education, secondary education, sports and physical education; the National Youth Council of Cameroon, churches and administrative authorities, community health workers, local radio station, and the Peace Corps Cameroon volunteer. Five days of awareness and voluntary, free and anonymous HIV testing in schools, churches, market squares and car station; a 10km marathon and an inter-school football game against stigma, a two-day workshop on "Gender and HIV / AIDS-Men as Partners" were implemented. The screening flow saw a collective pre-counselling, electronic identification, testing, demonstration on the use of condoms, post-test individual counselling. The data were collected by the Comm Care app. A descriptive analysis of clients allowed us to identify hotspots for screening young people. This project received financial support from PEPFAR and material support from local partners.

## Results

We screened 2024 clients, including 1623 (80%) aged ≤ 35, 994 (49%) men and 1030 (51%) women. The number of young people at their first ever HIV test was 1256 (77%). The proportion of youths in the general population who attended screening sites were 831 (97%) in the Schools, 212 (78%) at the motor park after the marathon, 159 (65%) at the exit of mass from the Church Catholic, 421 (64%) at the market square. Around 322 (20%) young people were currently out of school, from whom all the 4 (0.2%) who tested HIV-positive. Nine participants were tested HIV positive and linked to HIV care and treatment services. We also distributed over 20000 condoms, disseminated critical information on HIV transmission, prevention and treatment by distributing laminated HIV Educational Info Cards. These cards also indicated local HIV services available.

## Discussion

Integrating HIV testing into an action package for young people and with young people is feasible and effective in rural areas. Using Information and Communication Technology (ICT) for electronic data collection during mass testing is feasible and fast. Testing is also feasible in the church or religious setting for the young (Catholics) faithful but condom distribution was not allowed by the Catholic church leaders. The project has enabled 77% of young people to know their HIV status for the very first time in their lives. Schools are the easiest to reach sites but out-of-school youths are likely to be at a higher risk of HIV infection. Low HIV prevalence rate among clients may be due to the healthy volunteer effect where people at high risk or those with known HIV positive status do not often participate in voluntary testing. We recommend HIV programmes to be inspired by this example to improve HIV testing amongst young people. However, issues related to coordination and cost should be addressed during planning.

## Conclusion

This pilot study has demonstrated that ongoing youth development programmes in Cameroon can provide opportunities to insert HIV testing for young people and thus improve their HIV status awareness.

### What is known about this topic

- HIV testing is low among young people especially in sub Saharan Africa;
- Services and interventions to accelerate HIV testing among youths are available but enormous barriers do exist;
- According to the National Strategic Plan for HIV/AIDS in Cameroon 2014-2017, the Ministry of Health aimed for 80% of key populations to know their HIV status but gave no clear strategy on how to target youth for HIV testing.

### What this study adds

- Through the inclusion of youth friendly HIV testing within a larger HIV educational program-this project was able to break down barriers of access for youth to essential HIV services;
- This project's strategy of integration and multisectoral collaboration has succeeded in reaching most young people with HIV testing;
- Youth friendly interventions can be led by youths themselves with support from their leaders and services dedicated to them.
